# Opto-electrical bimodal recording of neural activity in awake head-restrained mice

**DOI:** 10.1038/s41598-021-04365-7

**Published:** 2022-01-14

**Authors:** Luis Fernando Cobar, Alireza Kashef, Krishnashish Bose, Ayumu Tashiro

**Affiliations:** School of Biological Sciences, Nanyang Technological University, Singapore, Singapore

**Keywords:** Optical imaging, Neuroscience, Extracellular recording

## Abstract

Electrical and optical monitoring of neural activity is major approaches for studying brain functions. Each has its own set of advantages and disadvantages, such as the ability to determine cell types and temporal resolution. Although opto-electrical bimodal recording is beneficial by enabling us to exploit the strength of both approaches, it has not been widely used. In this study, we devised three methods of bimodal recording from a deep brain structure in awake head-fixed mice by chronically implanting a gradient-index (GRIN) lens and electrodes. First, we attached four stainless steel electrodes to the side of a GRIN lens and implanted them in a mouse expressing GCaMP6f in astrocytes. We simultaneously recorded local field potential (LFP) and GCaMP6f signal in astrocytes in the hippocampal CA1 area. Second, implanting a silicon probe electrode mounted on a custom-made microdrive within the focal volume of a GRIN lens, we performed bimodal recording in the CA1 area. We monitored LFP and fluorescent changes of GCaMP6s-expressing neurons in the CA1. Third, we designed a 3D-printed scaffold to serve as a microdrive for a silicon probe and a holder for a GRIN lens. This scaffold simplifies the implantation process and makes it easier to place the lens and probe accurately. Using this method, we recorded single unit activity and LFP electrically and GCaMP6f signals of single neurons optically. Thus, we show that these opto-electrical bimodal recording methods using a GRIN lens and electrodes are viable approaches in awake head-fixed mice.

## Introduction

Neural activity of the brain has been conventionally investigated by electrical recording and optical imaging^1^. Electrical recording has the advantage of high temporal resolution, which allows us to detect action potentials of individual neurons and high frequency neural oscillations reflecting the functional state of a local network^2–7^. Optical imaging using calcium indicators has the advantage of simultaneous recording of a large number of cells^8,9^, although the temporal resolution is low due to the chemical properties of currently available calcium indicators. In addition, using cell-type-specific expression of genetically encoded indicators, the activity of different cell types can be determined while it is not straightforward to do it when performing electrical recording in behaving animals.

Using either electrical or optical recording with its own set of advantages, many studies have contributed to our understanding of the brain functions. However, there are some circumstances where it would be beneficial to merge their advantages and perform opto-electrical bimodal recording. For example, one can monitor different functional states of the brain using electrical recording of local field potential (LFP) and simultaneously characterize the activity of a large number of a specific cell type using calcium imaging^10,11^. Another example would be to characterize the relationship between neuronal activity, which can be monitored electrically, and the activity of astrocytes, which requires calcium imaging. Thus, opto-electrical bimodal recording is important to advance our understanding of the brain functions.

**Figure 1:**
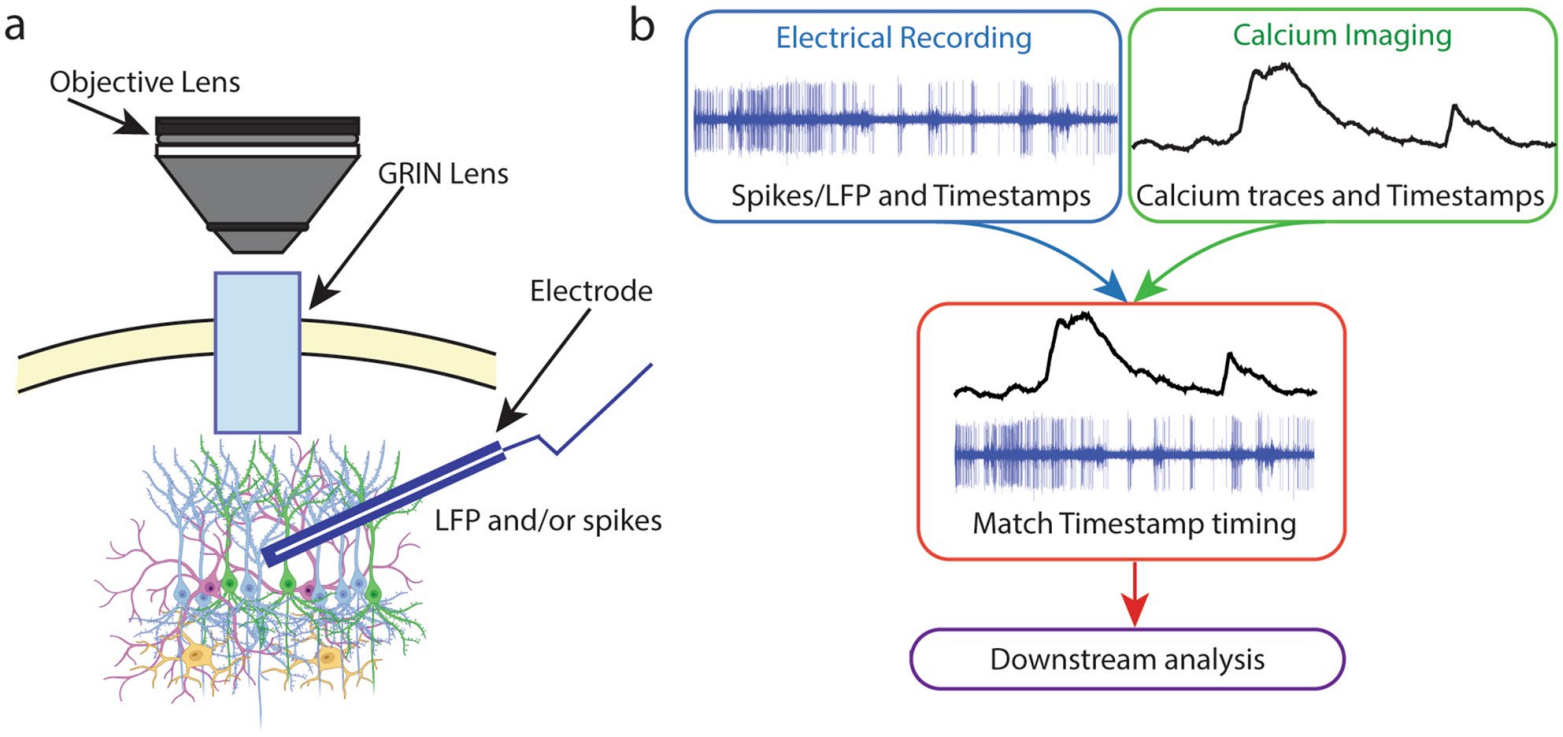
Opto-electrical bimodal recording. (**a**) Schematic of our experimental approach of opto-electrical bimodal recording. (**b**) A flow chart of processing bimodal recording data for downstream analysis.

In this study, we devised methods for opto-electrical recording by combining calcium imaging through a gradient-index (GRIN) lens and electrical recording using different types of electrodes. By chronically implanting the GRIN lens and electrodes in proximity, our method allows for simultaneous opto-electrical monitoring from the same brain area in awake head-fixed mice (Fig. 1). In such bimodal recording, one main challenge is the precise placement of the lens and electrode. To simplify that, we designed a custom-made 3D printed scaffold which ensures the accurate placement of a GRIN lens and an electrode. We validated the usefulness of this scaffold in chronic bimodal recording in an awake, behaving mouse. This method also allows for subsequent recovery and re-using of the lens and electrode to make it cost-efficient. We present three examples of opto-electrical bimodal recording to provide the ideas of how these methods can be implemented in different lines of studies.

## Results

**Figure 2:**
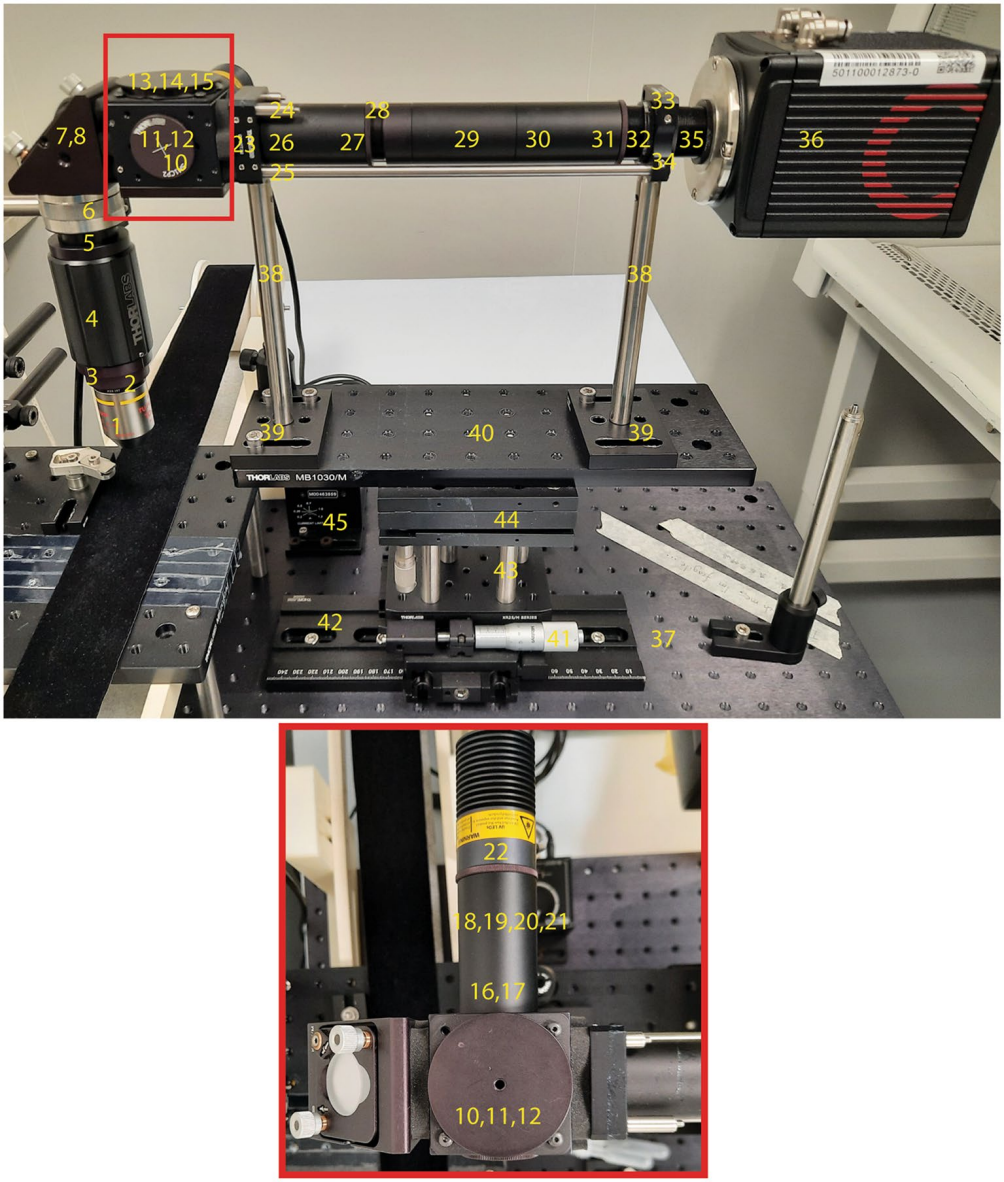
Microscope assembly used for bimodal recording. (Top) Numbers indicate corresponding description of parts in Table 1. (Bottom) A magnified top view of the red square in top.

We show three methods of bimodal recording, in each of which we combined optical imaging through a GRIN lens and a custom-made microscope (Fig. 2) with different ways to record electrical activity. For each approach, we show an example recording result.

### Bimodal recording: LFP and astrocytic calcium activity

**Figure 3:**
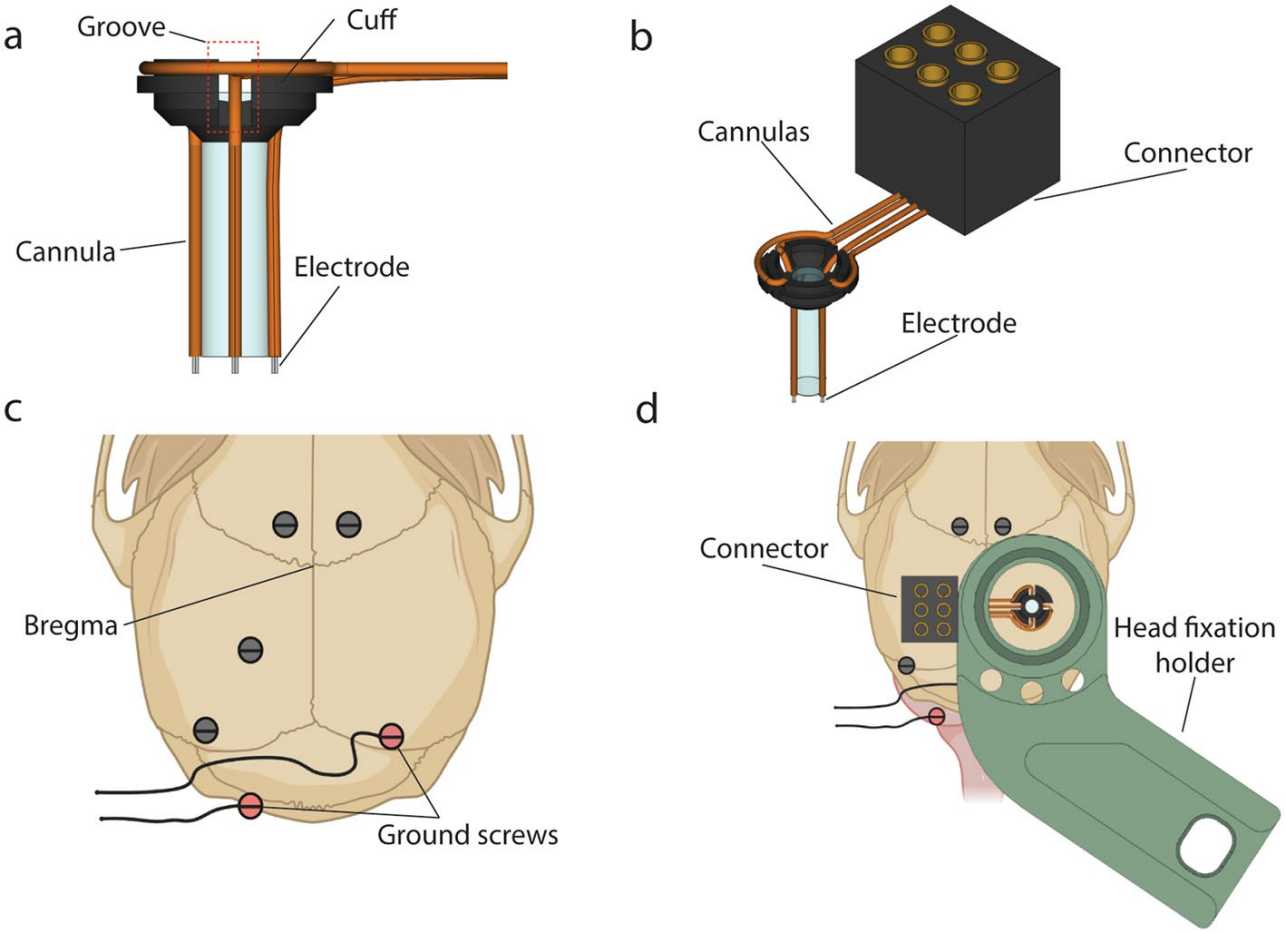
GRIN lens-stainless wire electrode assembly and its implantation on the mouse skull, used for Fig. 4. (**a**) Side view of a lens-electrode assembly (**b**) The lens-electrode assembly with a connector. (**c**) Positioning of screws and ground screws on the mouse skull. (**d**) Position of lens-electrode assembly and head fixation holder over the mouse skull.

The first approach is the simplest and uses a set of four insulated stainless steel wire electrodes secured to the side of a GRIN lens (Fig. 3). Each electrode was placed inside a flexible polyimide tubing cannula. The tips of the electrodes were extended by approximately 150 µm from the bottom surface of the GRIN lens. The working distance of the GRIN lens (from its bottom surface to a focal plane) is 100–300 µm. Therefore, in this arrangement, we can record optical and electrical signals from similar depth below the GRIN lens.

**Figure 4:**
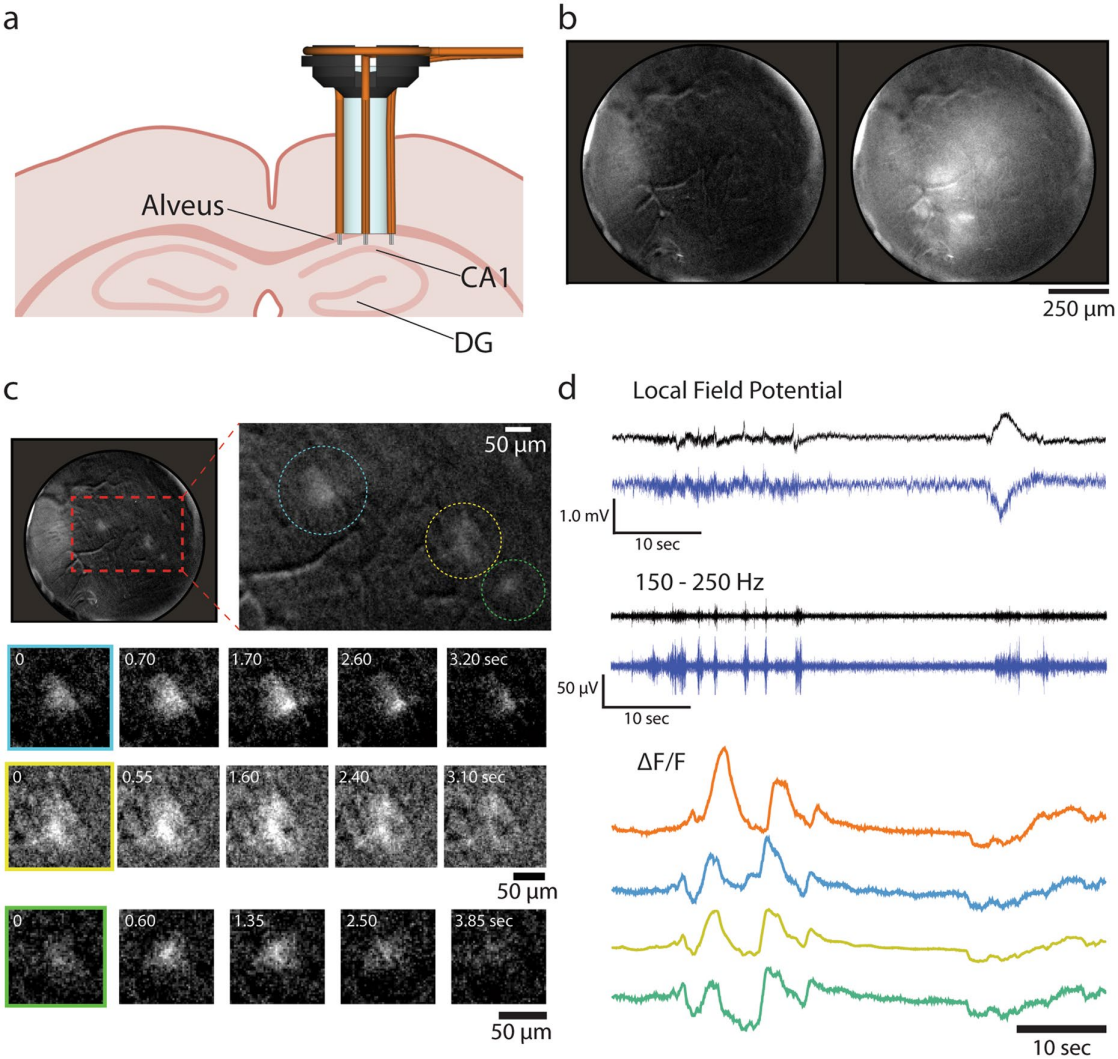
Bimodal recording of astrocyte activity and LFP in the CA1 area using a GRIN lens-wire electrode assembly. (**a**) Position of an electrode-lens assembly in the mouse brain. (**b**) Large, dispersed calcium signalling events. Left: A frame before a large calcium event. Right: A frame at the peak of a large calcium event. Most of the field of view also shows changes in fluorescence, potentially involving the coordinated increase in intracellular calcium concentration of multiple astrocytes that were beyond the focal plane. The circular edge in the images corresponds to the edge of the GRIN lens. (**c**) Calcium signalling events in individual astrocytes. Top, a frame showing three astrocytes exhibiting a calcium event simultaneously. Bottom, magnified images of the three astrocytes (blue, yellow and green, corresponding in the Top right image) over five time points. (**d**) Bimodal recording of LFP and astrocytic calcium signalling activity in the CA1 area. Electrical and optical signal traces are temporally matched. All images and traces in this figure were taken 1 week after electrode and lens implantation surgery.

Using this bimodal recording method, we imaged calcium signalling activity of astrocytes while electrically recording LFP at the same time. We selectively expressed genetically encoded calcium indicator GCaMP6f in astrocytes by injecting a viral vector containing a regulatory element from the GFAP gene. One week later, we implanted the GRIN lens-electrode assembly in a way that its bottom surface was immediately above the aspirated surface over the CA1 (Fig. 4a) so that we could perform bimodal recording from the CA1 pyramidal cell layer. After another week, we observed fluorescence changes in large, dispersed area, which was probably from astrocytes out of the focal plane (Fig. 4b). We also observed that well-defined individual astrocytes showed clear changes in GCaMP6f fluorescence (Fig. 4c: blue, yellow, green).

While the optical recordings were performed, we also monitored LFP through the four implanted electrodes in the CA1 area. We detected typical patterns of neural activity in the CA1 area such as sharp waves and ripples, as clearly shown in band-pass filtered traces in Fig. 4d (150–250 Hz). By comparing the simultaneously recorded bimodal signals, we were able to examine the correspondence between the calcium dynamics in astrocytes and different activity patterns in the LFP (Fig. 4d).

### Bimodal recording: LFP and neuronal calcium activity in CA1 pyramidal cells

**Figure 5:**
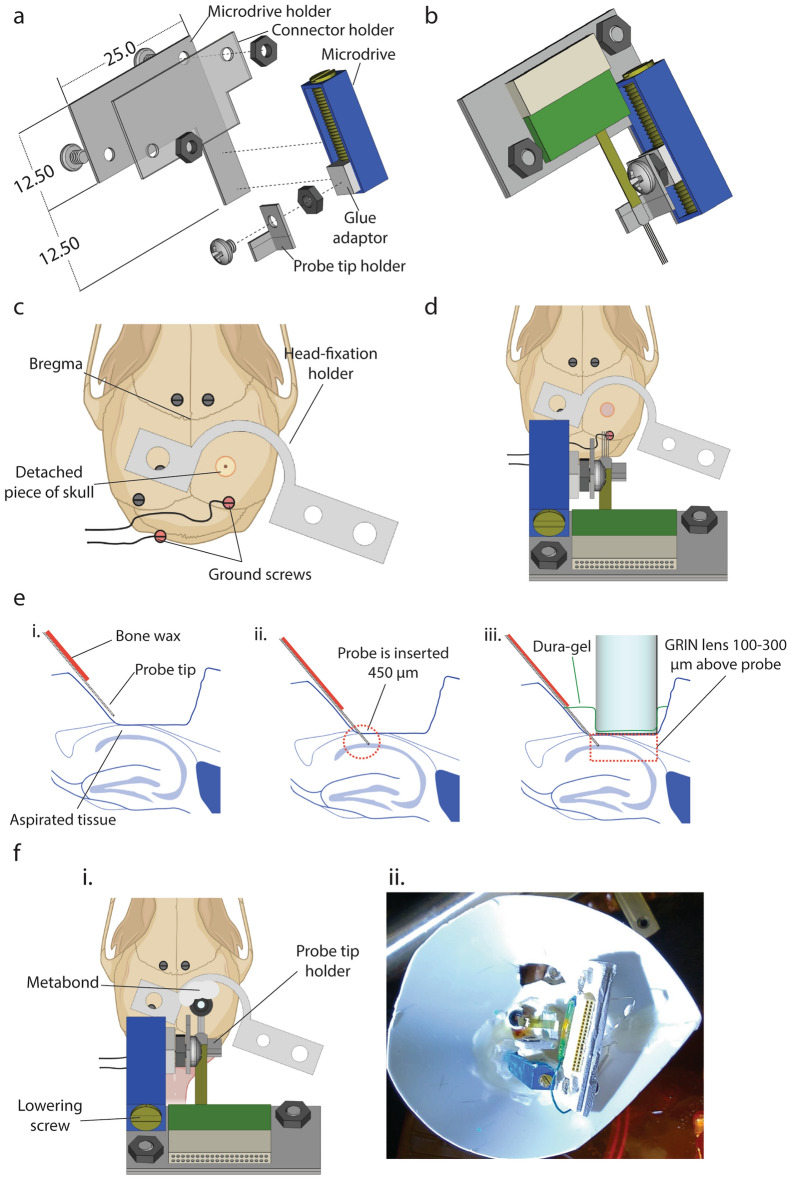
Microdrive assembly and its implantation together with a GRIN lens, used for Fig. 6. (**a**) Parts of a microdrive assembly. Dimensions are described in mm. (**b**) Completed microdrive assembly. (**c**) Positioning of screws, ground screws and head-fixation holder on the mouse skull. (**d**) Positioning of the microdrive assembly on the skull before inserting the tip of silicon probe into brain tissue. (**e**) Schematic showing implantation procedure from the sagittal view of the hippocampus. i. The silicon probe is lowered into the aspirated space under the skull. ii. The silicon probe is lowered 450 µm into the brain. iii. A GRIN lens is lowered closed to the aspirated surface of brain tissue and placed as closely as possible to the probe tip. We covered exposed surfaces of the aspirated brain tissue with Dura-gel. (**f**) i. Final position of the microdrive-lens assembly on the mouse skull. ii. A custom-made cover protecting the microdrive-lens assembly on the mouse skull.

In the second approach, we combined a GRIN lens and a silicon probe (Fig. 5). We made a custom-made microdrive and mounted a silicon probe on it. In this way, we were able to move the silicon probe deeper into the brain after it was chronically implanted. We prepared the GRIN lens and the microdrive-probe assembly separately and implanted them together in a surgery (See Materials and Methods; Fig. 5).

**Figure 6:**
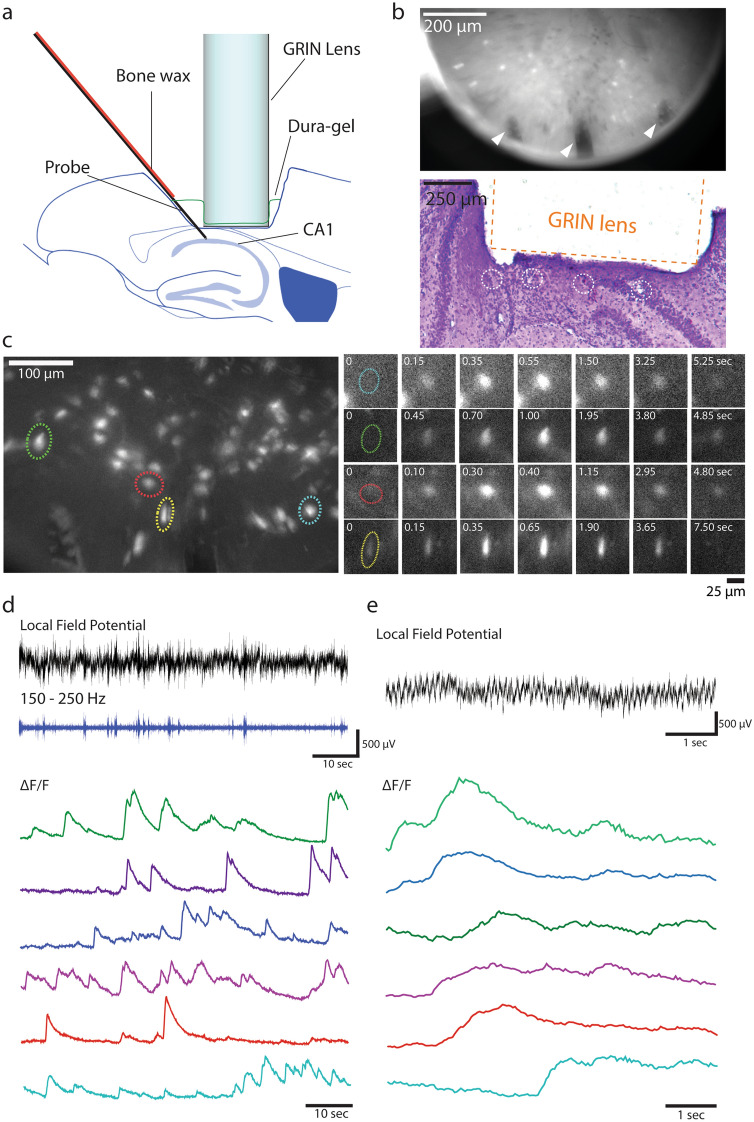
Bimodal recording of neuronal calcium signalling and LFP in CA1 using a GRIN lens and a silicon probe. (**a**) Position of a GRIN lens and a silicon probe for bimodal recordings in the CA1 area. (**b**) Confirmation of the placement of the GRIN lens and the silicon probe. Top: A frame of fluorescence image showing the existence of silicon probe shanks under the GRIN lens (Arrowheads). Bottom: An image of a cresyl violet-stained brain section showing the location of the four shanks of the silicon probe (white dotted circles). Orange dashed line indicates the estimated position of the GRIN lens. (**c**) Left: A ΔF/F image showing Individual neurons expressing GCaMP6s. Right: Example frames indicating changes in fluorescence during a calcium event for each of four neurons. Colors correspond to colors of dashed circles in the left image. (**d**) An example of temporally matched traces of LFP and neuronal calcium signals. Raw and band pass-filtered traces (150–250 Hz) are presented in black and blue lines, respectively. The band pass-filtered traces show multiple episodes of sharp wave-associated ripples. Calcium signals from different neurons are presented in different colours. (**e**) Another example temporally matched traces while the mouse runs. We observed clear theta oscillations (8–12 Hz). Data in this figure were recorded 32 days after implantation.

Using this approach, we imaged calcium signalling activity in neurons and electrically recorded LFP in the CA1 area (Fig. 6). For this purpose, we first injected a mouse with a viral vector containing the regulatory element from the CaMKIIα gene to express GCaMP6s in neurons in the CA1 area. One week later (day 0), we chronically implanted the mouse with a GRIN lens above the CA1 area and placed a silicon probe, attached to a custom-made microdrive assembly, immediately below the lens (Fig. 6a). We performed bimodal recording for > 1 month after implantation. During the recording sessions, we head-fixed the mouse above a running wheel; the mouse had periods of running and immobility.

On day 12, we performed bimodal recording. By this recording session, we had advanced the silicon probe ~ 500 µm forward from the surface of aspirated brain tissue along the longitudinal axis of the shanks. We found three evenly spaced dark areas near the edge of the GRIN lens (Fig. 6b, top), which indicates the existence of three silicon probe shanks closely under the GRIN lens. The shanks continued to be visible until day 34 (the last recording session performed in the awake mouse) and day 39 (the last recording session performed under anaesthesia). We confirmed the location of the GRIN lens and silicon probe shanks in brain sections prepared after we completed the experiments (Fig. 6b, bottom). Three shanks were located ~ 100 µm or less below the bottom surface of GRIN lens. Another one was located around the edge of the GRIN lens; this seems to be why we observed only three of the four shanks through the GRIN lens.

We observed many GCaMP6s-expressing neurons (Fig. 6c,d, Supplementary video 1). We recorded calcium signalling activity, starting from day 12 until we performed the last recording session on day 39. The GCaMP6s signals in neurons had clear boundaries corresponding to their cell bodies (Fig. 6c). We observed calcium events more frequently in individual neurons (Fig. 6d) than in astrocytes (Fig. 4). These events showed relatively sharp rise and slower decay and lasted for up to several seconds (Fig. 6d), which are typical features of GCaMP6s fluorescence changes in neurons.

Using the silicon probe, we monitored LFP. On the day of implantation, we advanced the silicon probe tips 450 µm forward from the surface of aspirated brain tissue along the longitudinal axis of the shanks. We detected sharp waves, which is a typical electrophysiological feature in the hippocampal CA1 area. We continued to observe sharp waves and/or sharp-wave-associated ripples until the last day of experiment (Fig. 6d).

Data shown in Fig. 6c–e are an example of bimodal recording on day 32. We were able to compare local field potential and calcium signalling activity in individual neurons, for example, whether or not the occurrence of calcium signalling events in individual neurons corresponds to the period around sharp-wave-ripples (Fig. 6d). Theta oscillations were observed while the mouse was running on the running wheel (Fig. 6e), during which some of neurons showed calcium events. Although it is not in the scope of our current study, it would be interesting to examine how LFP oscillations at different frequency ranges correspond to calcium events in neurons in the CA1 area.

Thus, we successfully performed chronic bimodal recording of LFP and neuronal calcium signalling in the same brain area in awake, behaving mice.

### Bimodal recording: single-unit/neuronal calcium with a custom-designed 3D Printed scaffold

Whereas we managed to accurately place a silicon probe under the GRIN lens, we also found it challenging to consistently achieve the precise placement of the two separate components together during the surgery. Indeed, we experienced some failures in the precise placement of electrodes under a GRIN lens in test experiments that we did not describe in Fig. 6. To insert a silicon probe into the brain tissue at as a close position to a GRIN lens as possible, we needed to move the silicon probe while visually tracking its position under a stereomicroscope. The insertion site was inside an aspirated hole made in the brain tissue through craniotomy. Therefore, it was difficult for us to visually determine the insertion site immediately next to the GRIN lens.

**Figure 7:**
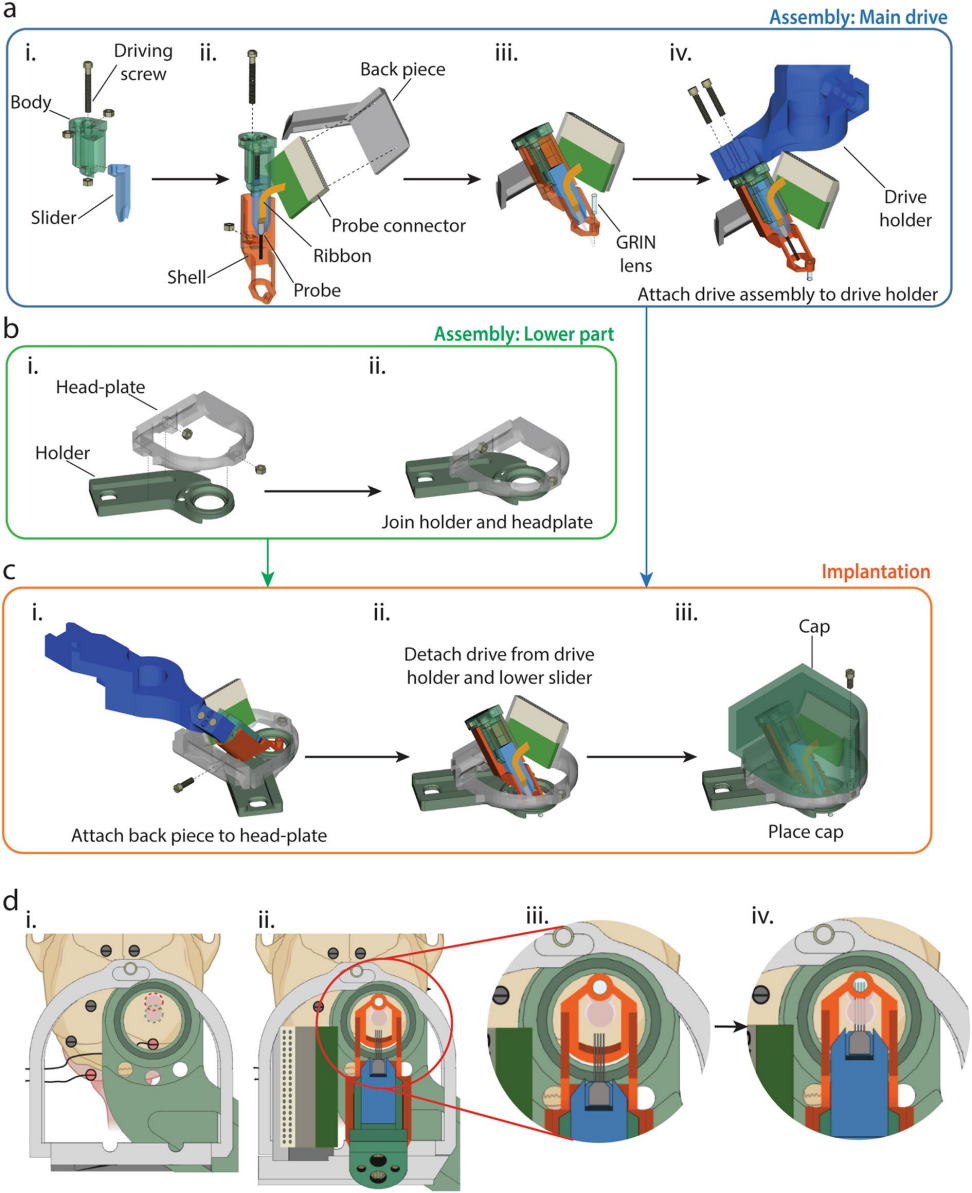
3D printed scaffold for a GRIN lens and a silicon probe and its implantation on the mouse skull. (**a**) Assembly of a scaffold. i. The slider and body are held together via a screw and a nut. Two extra nuts are placed in the body to later attach the completed scaffold to the drive holder in iv. ii. Attachment of the probe tip and connector to the slider and back piece, respectively using the Metabond glue. The shell and the slider + body are held together via another set of a screw and a nut. iii. Mounting of a GRIN lens on the shell. iv. Attachment of the scaffold to a drive holder for implantation. (**b**) Assembly of lower part. i. a head-plate and head-fixation holder. ii. Fixation of the two pieces with glue. (**c**) Implantation and protection by cover. i. Positioning of the scaffold using a stereotaxic manipulator. ii, Release of the scaffold from the drive holder after implantation. iii, Protection by cover after surgery. (**d**) Diagram for the implantation of the 3D printed scaffold. i. Positioning of the head plate and craniotomy. Two overlapping circular craniotomies (Red and green). ii. Positioning of the scaffold on the skull. The GRIN lens is inserted into the anterior craniotomy. iii. Magnified view of ii. iv. Insertion of the tip of the silicon probe into the craniotomy.

In view of these challenges, we set out to devise the third approach that makes it easier to implant a silicon probe electrode and a GRIN lens precisely and minimize the variability from one implantation to another. For this purpose, we developed a 3D printed scaffold to hold a silicon probe and a GRIN lens together. We started this effort by adopting a microdrive assembly designed by Chung et al.^12^ and modified it to accommodate a GRIN lens and other specific requirement for our purpose (Fig. 7).

This scaffold is composed of three main pieces: “slider”, “body”, and “shell” (Fig. 7a). The scaffold houses a GRIN lens and a silicon probe and works as a microdrive moving the electrode into the brain. The scaffold is attached to a backpiece and a head-plate (Fig. 7b). The backpiece and head-plate accommodate a connector for the silicon probe and a cap. The cap covers the scaffold and protects it from mechanical damage while a mouse is in its home cage. It can be detached during experiments (Fig. 7c). The scaffold and connecter are placed at an angle so that they do not block the access of an objective lens to the GRIN lens. The entire assembly is secured to a head fixation holder, which we use to fix the mouse head under the microscope (Fig. 7b). The whole assembly is lightweight (6.8 g) and does not affect the mobility of the mouse to engage in its normal activity inside or outside of the home cage.

**Figure 8:**
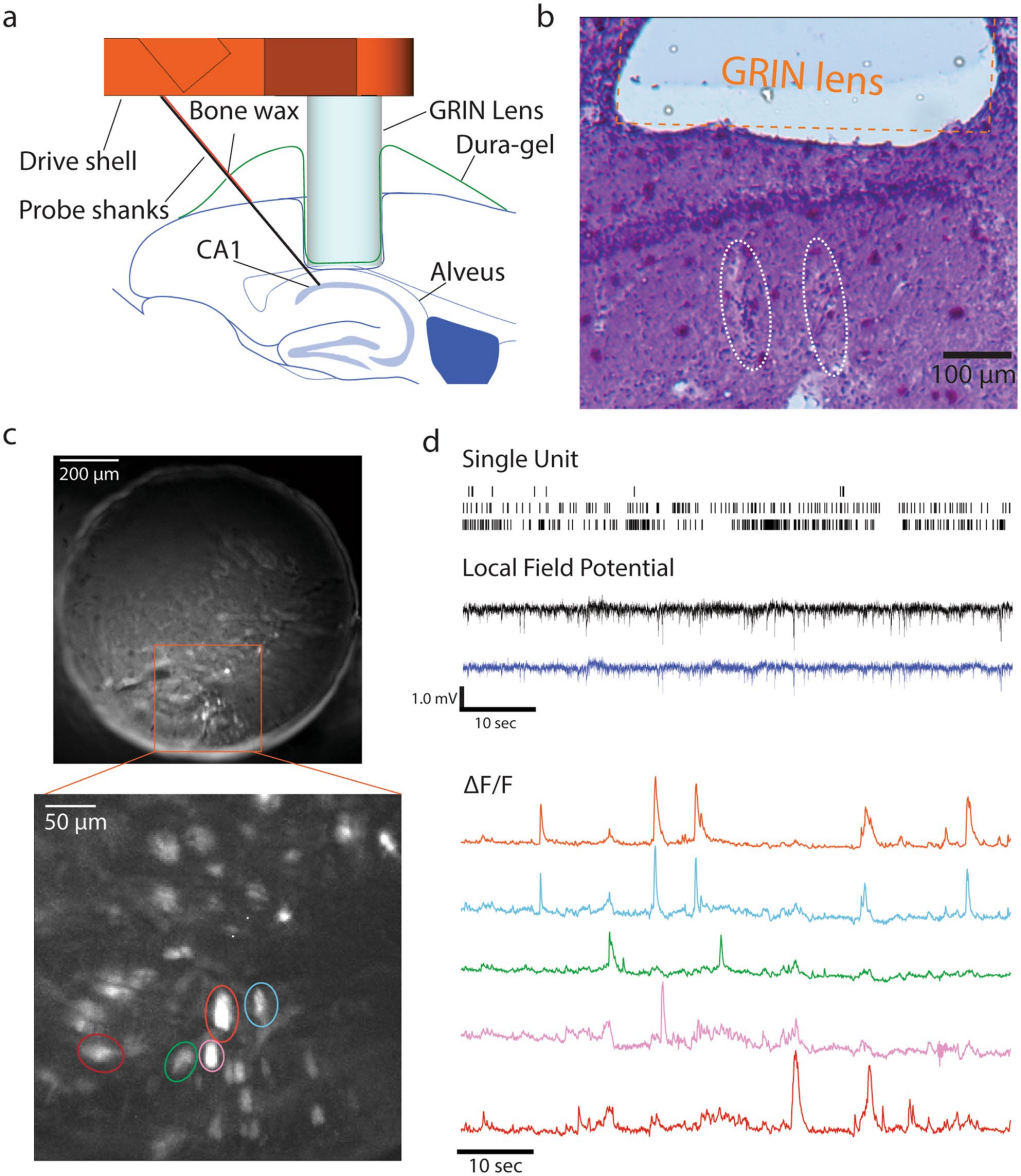
Bimodal recording of LFP, single unit activity and neuronal calcium signalling in the CA1 area using a 3D printed scaffold. (**a**) Diagram for the implanted positions of the 3D printed scaffold relative to the mouse brain. (**b**) An image of a cresyl violet-stained brain section showing the traces of the GRIN lens and two shanks of the silicon probe. Orange dashed line indicates the estimated edges of the implanted GRIN lens. Two traces of the shanks are indicated by white dashed ovals. (**c**) Top: A ΔF/F image of the entire GRIN lens in the scaffold. Bottom: A magnified ΔF/F image of the highlighted portion in the top image in the proximity of the tips of the recording probe. Individual cells are highlighted using coloured ellipses. (**d**) Top: Raster plots showing firing of three single units. Middle: Raw LFP signals from two different channels. Bottom: calcium signal traces of neurons highlighted by the same colors in the bottom image of (**c**). Data in (**c**,**d**) were recorded 4 weeks after implantation.

Using the scaffold, we performed calcium imaging, LFP and unit recording in the CA1 area of a Thy1-GCaMP6f mouse (Fig. 8a). During recording, we head-fixed the mouse on a treadmill. The mouse sometimes ran and sometimes stayed still. Using this method, we were able to place a silicon probe accurately within and/or slightly below the imaging volume of a GRIN lens, which we confirmed in brain sections prepared after we completed the bimodal recording experiments (Fig. 8b). We found the trace of the silicon probe shanks ~ 200–350 µm below the bottom surface of the GRIN lens.

On the day of implantation (day 0), we inserted the silicon probe into the brain tissue by rotating the driving screw for 1.5–2 turns. We detected sharp waves as well as spiking activity; however, the recording quality of spiking activity was not sufficient for spike sorting and we failed to isolate single unit activity. On day 15, we advanced the electrode 60 µm forward and performed the first bimodal recording. We detected sharp waves, ripples, and spiking activity (low quality again). In optical imaging, we detected dozens of neurons showing changes in GCaMP6f fluorescence. We continued to observe such neuronal calcium signalling activity throughout the period of the experiment. Figure 8c,d show an example result of bimodal recording on day 17. We succeeded in detecting single-unit spiking activity that were well isolated. Because these bimodal recording data may contain signals from the same individual neurons, we visually searched for a pair of electrical and optical neuronal data that showed a high degree of temporal matching. However, we did not find such an example in the limited number of sessions in this mouse. We continued to detect well-isolated single unit activity over several days. As expected, individual calcium signalling events visualized by GCaMP6f signals here were shorter compared with GCaMP6s signals in Fig. 6.

After we finished the experiment, we retracted the slider and withdrew the probe tip from the brain. Then we disconnected the slider and body from the shell by loosening the screw that had fixed them together. In this way, the assembly of the slider, body and silicon probe could be reused in next experiments. We intentionally designed the scaffold so that the recovery procedure would become easy.

Thus, using a 3D printed scaffold, we were able to chronically record LFP and single-unit activity electrically and simultaneously image GCaMP6f signals optically. This approach also simplifies the assembly, implantation and recovery of the recording probe.

## Discussion

In this study, we demonstrated the successful use of three different methods of opto-electrical bimodal recording of neural activity in awake mice. Placing simple wire electrodes or a more sophisticated silicon probe in the proximity of a GRIN lens, these methods allowed us to monitor calcium signalling activity in neurons or astrocytes while simultaneously recording LFP and/or neuronal spiking activity. Although many readers may not be familiar with building a microscope, the full description of required parts in Table 1 would be helpful for the first-time builders. The total cost of microscope components (excluding an objective lens and a camera) is ~ 3500 US dollars. Although we used a relatively high-end objective lens and camera, the use of lower-end models would be sufficient for many applications. Alternatively, an objective lens and a camera available in an existing fluorescence microscope can be used. Thus, our opto-electrical bimodal recording method in awake head-fixed mice is a viable option that is relatively low cost and easily implemented.

We designed our methods with the motivation to perform bimodal recording from the same area in the brain. Therefore, the accurate placement of the GRIN lens and electrodes is essential. In Fig. 4, we used the simple solution of attaching electrodes to the side of a GRIN lens. In the course of testing the second approach described in Figs. 5 and 6, we experienced a challenge in the precise placement of the silicon probe immediately below the GRIN lens. Precise placement is also important in terms of not damaging the expensive silicon probe, which is so fragile that the probe can be easily damaged by mistakenly hitting other components. This challenge motivated us to design a scaffold to accommodate a GRIN lens and a silicon probe together. As shown in Fig. 7, we designed and constructed the 3D-printed scaffold, which simplifies the assembly, implantation and recovery procedures and ensures the accurate placement of the lens and the probe.

For successful bimodal recording with a silicon probe (Figs. 6 and 8), it is important to achieve proper spatial arrangement among a GRIN lens, a silicon probe and a target brain area. We designed our implantation scheme with the following considerations. First, because we used the GRIN lens that has a working distance of ~ 100–300 µm, the GRIN lens needs to be implanted 100–300 µm above the target area. As shown in Figs. 6 and 8, we successfully placed the GRIN lenses in this distance range, although some portions of the pyramidal cell layer were found to be too close to or far from the GRIN lens in Fig. 7. Second, electrodes should be placed at some distance below the target brain area. This is because (1) electrical recording works from a distance (single-unit: ~ 50 µm, multi-unit: ~ 140 µm)^13^, (2) the close placement of electrodes causes physical damage of the target brain area and (3) the silicon probe shanks may block the optical imaging of cells below them. Considering that the target structure is above the shanks, we implanted the silicon probe in a way that its electrodes face upward, thereby being closer to the neurons above them. Most recording sites estimated from histological analysis both in Figs. 6 and 8 were located in the position that we aimed at, although a few of the four shanks in each case were off. We observed the shanks of the silicon probe through the GRIN lens in Fig. 6, but not in Fig. 8. This difference is likely due to their difference in distances between the GRIN lens and the shanks; the shanks in Fig. 8 seem to have entered the volume below the GRIN lens more deeply than in Fig. 6. Although it is helpful to be able to confirm the position of electrodes during the experiment, the positioning of the shanks too closely to the GRIN lens may not be ideal because of tissue damage as mentioned above.

We have presented our successful bimodal recording of LFP and neuronal/astrocytic calcium signalling. LFP provides critical information to define how a local neuronal network is functioning. Bimodal recording would be an essential tool for investigating how the functional states of a local neuronal network modulate astrocyte calcium signalling. For example, the involvement of astrocytes has been suggested in the generation of sharp waves and ripples^14^ and theta oscillations^15^ in the hippocampus. Further investigation of such possibilities requires to characterize how astrocytes are activated during sharp waves and ripples or theta oscillations; therefore, bimodal recording is critical for such studies. In addition, it has been suggested that adult neurogenesis in the dentate gyrus is regulated by local circuit activity^16–18^. Bimodal recording of local neuronal activity/LFP and calcium signalling in radial glia/neural stem cells would help understand how neural activity regulates adult neurogenesis. Furthermore, optical imaging allows us to reveal spatial distribution/pattern of activated neurons in a brain region of interest. Bimodal recording can be applied to determine how such spatial characteristics of local circuit activation are associated with LFP-defined functional states.

One potential application of this approach is to perform bimodal recording of the same individual neurons. To achieve it, we need to temporally compare the electrical and optical recording data from individual neurons and identify a pair of individual neuronal data that show a good match in the timings of their firing and calcium events. In this way, one can optically identify neuronal types (if a genetically-encoded indicator is expressed in a specific neuronal type) and electrically record neuronal spikes representing individual action potentials. In addition, it is of importance to clarify whether calcium signalling events and action potentials have one-to-one correspondence or if not what patterns of action potential firing result in calcium signalling events. In this study, we only imaged green fluorescence of GCaMP6s and GCaMP6f using a GFP/FITC filter set. Optical imaging for a different wavelength can be implemented by using an appropriate LED light source and a corresponding filter set. The use of dual band filters, multi-wavelength light source (e.g. a mercury lamp) and a color camera allows us to perform simultaneous dual color imaging. This approach would enable, for example, to monitor activity of cell populations that express different fluorescent tags in the same brain region. For example, simultaneous imaging of GCaMP and RCaMP signals together with electrical recording is feasible. Fluorescence energy transfer (FRET) imaging can be implemented using an appropriate filter set. While we used a low-cost, custom-made microscope, our bimodal recording approaches can be combined with more sophisticated imaging hardware such as two-photon microscopy^19^.

Another potential application of this approach lies in the use of new genetically encoded fluorescent probes which can monitor a wide variety of molecules involved in multiple brain processes, such as GABA^20^, norepinephrine^21^, ATP^22^, and Dopamine^23^. Because these newly developed tools have excitation/emission spectra similar to that of GFP, little to no modification would be necessary for the present approach to investigate the relationship among neuronal spiking activity, LFP patterns, and the temporal dynamics of synaptic release of specific neurotransmitters or other brain processes.

In conclusion, we devised three opto-electrical bimodal recording approaches to target a deep brain structure using electrodes and GRIN lens and demonstrated that these three bimodal recording methods are viable approaches in awake head-fixed mice. This approach is affordable and versatile and can be adapted to a wide variety of setups for different lines of research, by harnessing the strengths of both electrical and optical recording.

## Materials and methods

### Microscope assembly

We built a custom-made microscope (Fig. 2) (Pierce et al., 2010). Table 1 lists parts of the microscope. We designed the microscope in a way that an objective lens can be precisely moved in the X and Y axes and for tilt. The objective lens faces downwards to acquire images through a GRIN lens implanted vertically on the skull of a head-fixed mouse. Excitation light was provided by a power-adjustable, light-emitting diode (470 nm, 650 mW mounted LED, Thorlabs Inc., New Jersey, United States). Fluorescence images were captured by an ORCA-Flash4.0 V3 C13440-20CU Digital CMOS Camera (Hamamatsu Photonics, Iwata, Japan) and HCImage live software (version 4.6.1.3, Hamamatsu Photonics, Iwata, Japan, https://hcimage.com/) through an emission filter (central wavelength: 530 nm, band width: 43 nm).

**Table 1:** Material list for microscope assembly.

Part description	Position code	Purpose	Manufacturer	Part number
20 × infinity corrected objective lens, f = 200 mm, NA = 0.45, WD = 8.2 mm	1	For 20 × optical magnification	Nikon	CFI S Plan Fluor ELWD 20XC
Adapter with external SM1 threads and internal M25 × 0.75 threads	2	For using Nikon objective	Thorlabs	SM1A12
SM1 Lens tube 2.00"	3	To be placed inside zoom housing	Thorlabs	SM1L20
SM1 Zoom Housing for Φ1" Optics, Non-rotating, 2" (50.8 mm) travel	4	For coarse focussing	Thorlabs	SM1NR1
SM1 Lens tube 0.5"	5	Connecting two focusing tubes	Thorlabs	SM1L05
SM1 Zoom Housing for Φ1" Optics, Non-rotating, 4.1 mm travel	6	For fine focussing	Thorlabs	SM1ZM
Φ1" (Φ25.4 mm) Protected aluminium mirror	7	Right-angled placement of any optical component	Thorlabs	PF10-03-G01
Right-angle Kinematic mirror mount	8	Tip/tilt adjustment of plane mirror, for adjustment of mirror mount to filter cube	Thorlabs	KCB1/M
Cage assembly rod, 1" Long, Φ6 mm–2 pieces	9	For top attachment of mirror mount of filter cube	Thorlabs	ER1-P4
Blank cover plate	10	For closing filter cube	Thorlabs	B1C/M
Externally SM1-Threaded End cap	11	For closing filter cube	Thorlabs	SM1CP2
30 mm Cage Cube, Φ6 mm through holes	12	For placement of beam splitter and attaching LED to microscope	Thorlabs	C6W
Fixed cage cube platform for C4W/C6W, metric	13	Platform for beam splitter	Thorlabs	B3C/M
Φ1" Optic mount for 30 mm cage cube	14	For mounting the beam splitter	Thorlabs	B5C1
1" Longpass Dichroci Mirror, Transmission (> 90%): 520–800 nm / Reflection (> 95%): 380–490 nm	15	Splitting/merging of excitation and emission light in the same optical path	Thorlabs	DMLP505
GFP Excitation Filter, CWL = 469 nm, BW = 35 nm	16	Suitable for selective transmission of light in the range of 450–487 nm	Thorlabs	MF469-35
SM1 Lens Tube, 2.00"	17	For housing excitation filter (Can be used for housing 1" condenser lens)	Thorlabs	SM1L20
SM1 Coupler, external Threads, 0.5" Long	18	For connecting different tubes	Thorlabs	SM1T2
SM1 Lens Tube, 1.00"	19	For housing the 0.5" condenser lens	Thorlabs	SM1L10
Adapter with external SM1 threads and internal SM05 Threads, 0.40" Thick	20	For housing the 0.5" condenser lens in the SM1 tube	Thorlabs	SM1A6T
Aspheric Condenser Lens Φ1/2", f = 8 mm, NA = 0.78, ARC: 350–700 nm	21	Collimation of LED	Thorlabs	ACl12708U-A
470 nm, 650 mW (Min) Mounted LED	22	Excitation light source	Thorlabs	M470L3
SM1-Threaded 30 mm Cage Plate 0.50" Thick	23	For holding the filter cube, objective and connecting parts	Thorlabs	CP02T/M
Cage assembly rod, 2" Long, Φ6 mm–2 pieces	24	For top attachment of filter cube to cage plate	Thorlabs	ER2
Cage assembly rod, 10" Long, Φ6 mm–2 pieces	25	For top attachment of filter cube to cage plate and camera	Thorlabs	ER10
f = 250.0 mm, Φ1" Achromatic Doublet, ARC: 400–700 nm	26	For focussing infinity corrected image on the camera sensor	Thorlabs	AC254-250-A
SM1 Lens Tube, 2.00"	27	For housing the focussing lens	Thorlabs	SM1L20
Φ1" Adjustable Lens Tube, 1.31" Travel Range	28	Makes it possible to easily take out and replace any of the 3 SM1 tubes	Thorlabs	SM1V15
SM1 Lens Tube, 2.00"	29	For housing the focussing lens	Thorlabs	SM1L20
FITC Emission Filter, CWL = 530 nm, BW = 43 nm	30	For blocking unwanted light from reflection or auto fluorescence	Thorlabs	MF530-43
SM1 Lens Tube, 2.00"	31	For housing the emission filter	Thorlabs	SM1L20
SM1 Coupler, external Threads, 1" Long	32	For connecting SM1 tube to C-mount cage plate	Thorlabs	SM1T10
Adapter with External C-Mount Threads and Internal SM1 Threads	33	Adapter for SM1 tube to C-Mount cage plate	Thorlabs	SM1T10
C-Mount-Threaded 30 mm Cage Plate, 0.35" Thick	34	For supporting the weight of the camera	Thorlabs	CP13/M
C-Mount Coupler, External Threads, 1" Long	35	For connecting the camera to cage plate	Thorlabs	CMT10
ORCA-Flash4.0 V3 Digital CMOS Camera	36	For image capture	Hamamatsu Photonics	C13440-20CU
Aluminium Breadboard, 300 mm × 600 mm × 12.7 mm	37	For mounting the entire microscope	Thorlabs	MB3060/M
Φ12.7 mm Optical Post, SS, M4 Setscrew, M6 Tap, L = 100 mm	38	Height adjusted for head-fixed imaging of mouse in the stereotaxic frame	Thorlabs	TR200/M
Mounting base, 50 mm × 75 mm × 10 mm	39	Mounting the post with proper alignment	Thorlabs	BA2/M-P5
Aluminium Breadboard, 100 mm × 300 mm × 12.7 mm	40	For mounting the microscope on movable stage	Thorlabs	MB1030/M
Preassembled XY 25 mm Translation Stage, Side micrometers	41	Precise XY positioning of the entire microscope	Thorlabs	XR25P-K1
Extended Dovetail Baseplate	42	For long-range coarse movement of microscope in 1 direction	Thorlabs	XR25DR
Φ12.7 mm Optical Post, SS, M6 Setscrew, M6 Tap, L = 75 mm	43	Height adjustment for head-fixed imaging of mouse in stereotaxic frame	Thorlabs	TR75/M-P5
Multi axis tilt platform	44	To align the microscope objective with an implanted fibre	Newport	M-37
T-Cube LED Driver	45	For controlling LED intensity	Thorlabs	LEDD1B

### Subjects

All experimental procedures involving live mice were performed according to NACLAR guidelines and the approval by the IACUC at Nanyang Technological University, and are reported in accordance with ARRIVE guidelines (https://arriveguidelines.org). We used C57Bl/6 mice for Figs. 4 and 6 and a Thy1-GCaMP6f transgenic mouse (C57BL/6 J-Tg(Thy1-GCaMP6f)GP5.17Dkim/J, the Jackson laboratory, USA) (Dana et al., 2014) for Fig. 8. All the mice used were male and approximately 4 months old at the time of the surgery. We housed all mice in transparent acrylic cages in a temperature and humidity-controlled environment (20–22 °C, 50–60% humidity) on a 12-h light/dark cycle with ad libitum access to food and water unless otherwise stated. After the surgery, we housed mice individually. We used water as a reward to motivate the mice to run on a treadmill under the microscope. For this purpose, we restricted the amount of water given to the mice starting 3 days before placing the mouse in a treadmill. We adjusted the volume of water given to the mice so that they ran well on the treadmill, while keeping at least 85% of ad libitum body weight.

### Lens-electrode assembly

For Fig. 4, we used a lens-electrode assembly consisting of a GRIN lens (1.0 mm diameter, 4.0 mm length, Inscopix, CA, USA) attached to four stainless steel (0.075 mm Φ. GoodFellow Cambridge Limited, England) wire electrodes at its side (Fig. 3a). We made four grooves on the cuff of the GRIN lens, through which four flexible polyimide tubing cannulas (0.20 mm Φ. MicroLimen, USA) passed. We attached cannulas to the side and cuff of the GRIN lens with glue (Loctite 401, Henkel Corporation, Germany). We removed insulation material from the tips of insulated stainless wires to use them as electrodes and inserted them individually into each cannula. We cut the wires so that the electrode tips extended ~ 150 µm from the bottom of the lens. The other ends of the wires were connected to the metal pins of a connector that was cut out from a VersaDrive 8 drive (Neuralynx, Inc., MT, USA) (Fig. 3b). In addition to the wires used as electrodes, two additional wires were connected to this modified connector, and we stripped their ends of the insulation. These wires were later soldered to the wires attached to ground screws to be used as reference electrodes.

For Fig. 6, we used a silicon probe electrode (Buzsaki 32, HC32_21mm, Neuronexus, MI, USA), which we attached to a custom-made microdrive assembly (Fig. 5a). We made the microdrive by cutting away a part of potentiometer (3006P Rectangular Multi-Turn Potentiometers, Vatronics Technologies Limited, USA), so that its screw was exposed (Grohrock et al., 1997). We applied the Metabond glue around the screw to make a small cube (we call it “a glue adaptor”), which moves when we rotate the screw; a full turn of the screw moves the adaptor ~ 300 µm. We attached a nut to the glue adaptor, to which we fixed a probe tip holder by another screw (Fig. 5a). We cut plastic plates to make a connector holder and microdrive holder (Fig. 5a), which we assembled together by two sets of nuts and bolts. We attached the microdrive to the microdrive holder (Fig. 5a) and glued the connector of a silicon probe electrode to the connector holder and the plastic part of the tip (but not the shanks or ribbon cable part) of the silicon probe to the probe tip holder (Fig. 5b). Before implantation, we cleaned the tips of the probe in a 10% solution of Decon 90 (Decon Laboratories, UK) in ddH_2_O for 90 min and then rinsed it with ddH_2_O. We confirmed that the channels in the probe were functional by measuring their impedance values using a NanoZ device (Neuralynx, Inc., MT, USA). The GRIN lens was not attached to the microdrive assembly and implanted separately as described below.

For Fig. 8, we used a custom-made 3D printed parts: a slider, a body, a shell, a backpiece, a headplate, a drive holder and a cap (Fig. 7). These parts were 3D-printed with Stereolithography technology by a commercial service (Engineering Computer Services (S) Pte Ltd). They used Figure 4 Standalone printer (3D systems, Inc) and Figure 4  Rigid Gray material, and washed the printed parts, removed support structures and cured the printed parts in a UV box. Computer-aided design files for the 3D-printed parts are available on GitHub (https://github.com/AyumuTashiro/bimodal).

We assembled the slider, the body and the shell to form a scaffold to carry a silicon probe and a GRIN lens. We attached the slider to the body via a 00–90 machine screw (9/16"), which we call a driving screw. We inserted the driving screw from the top of the body and into the screw hole of the slider (Fig. 7a,i). The end of the driving screw protruded out of the body. We screwed it into a nut until the nut touched the body, so that the driving screw was stably fixed in the body but was still able to smoothly rotate. We cut the portion of the driving screw that extended beyond the nut and fixed the screw and the nut by soldering them together. Rotation of the driving screw drives the movement of the slider; one full turn of the screw corresponds to 450 µm movement. We attached two other nuts to the top part of the body with glue (Fig. 7a,i). Later, we used these nuts to fix the scaffold to a drive holder when we fixed the scaffold to the mouse skull (Fig. 7a,iv). We connected the body to shell via another 00–90 machine screw (9/16") (Fig. 7a,ii). We inserted the screw from the top of the body and screwed it into a nut on the shell. We turned the driving screw until the slider reached the lowermost position. We then glued the probe tip to the slider and the connector to the backpiece (Fig. 7a,ii). To avoid placing the probe’s ribbon under stress, from this point onwards, we temporarily attached the backpiece and connector on the shell using a small amount of Blu tack. We removed the cuff of a GRIN lens and inserted the GRIN lens into the GRIN lens holding hole of the shell (Fig. 7a,iii). Under a stereomicroscope, we adjusted the position of the GRIN lens so that its bottom end was approximately 150 µm above the probe tips when we moved the slider all the way down. We fixed the GRIN lens to the shell, applying super glue to the point of contact between the GRIN lens and the shell while avoiding covering the surface of the lens. Before implantation, we measured impedance values using a NanoZ device and cleaned the probe using Decon 90, as described previously for Fig. 6. Parallel to the drive assembly, we attached two nuts to the head plate. (Fig. 7b,i) and glued a head plate and a head-fixation holder together (Fig. 7b,ii; https://hhmi.flintbox.com/technologies/b9aab117-9114-48e0-9d62-fc929ed30675).

### Virus injection

One week before the implantation surgery, we injected 1.0 µl of virus solution into the hippocampal CA1 area of mice [antero-posterior: 1.90 mm, medio-lateral: 1.40 mm from the bregma, dorso-ventral: 1.25 mm (for Fig. 4), 1.60 mm (for Fig. 6) from skull surface] at a rate of 0.2 µl/min using a syringe (Hamilton, Model 80100, Knurled Hub NDL) with a blunt ended needle (25 gauges, 2.75″/69.9 mm, point style 3; Hamilton, Reno, NV, USA). We used viral vectors expressing GCaMP6f under the control of the regulatory element from the GFAP gene (AAV5/pZac2.1 gfaABC1D-cyto-GCaMP6f, prepared by Addgene, a gift from Dr. Baljit Khakh, Addgene plasmid # 52925) for Fig. 4 and GCAMP6s under the control of the regulatory element from the CaMKIIα gene [prepared as previously described (Kitanishi et al., 2015)] for Fig. 6.

### Implantation of lens-electrode assembly

Deep anaesthesia was induced in mice with a mixture of 4% isoflurane and 2 L/min oxygen and then maintained at 1.5% isoflurane in 0.8L/min oxygen flow. Once anesthetized, we injected analgesic buprenorphine (0.1 mL per 10 g of body weight) and local anaesthetic lignocaine (0.05 mL at the incision site) subcutaneously. We shaved the scalp of the mice and mounted their heads in a stereotaxic frame (KOPF Instruments, CA, USA). We made a midline incision of the scalp approximately 2.0 cm long in the antero-posterior direction using a scalpel. We attached a drill (H.MH-17018 FOREDOM Electric Co, USA) with a drill bit (0.5 mm diameter, Fine Science Tools, Canada) to the stereotaxic frame on top of the bregma. Next, we moved the tip of the drill bit 1.9 mm posteriorly and 1.4 mm laterally over the right hemisphere. At this location, we drilled the skull to make a shallow mark where we later made craniotomy. Then, we drilled six holes on the skull (Fig. 3c) using the same drill bit and inserted one screw into each hole. Before insertion, we soldered two of the screws, referred to as “ground screws”, to stainless steel wires. We used these ground screws as the reference for the electrophysiological recording. We used a trephine (1.8 mm diameter, Fine Science Tools, Canada) to cut out a circular piece of the skull with a center at the position where we had marked. However, we left it in place while disconnected from the rest of skull. After this step, we performed different procedures for individual experiments, which we describe below individually.

For Fig. 4, we removed the disconnected piece of skull using a bent 25-gauge needle. Through the craniotomy, we aspirated the cortical tissue and made a cylindrical space which the GRIN lens fit in. For aspiration, we used a blunt-end, curved 25-gauge needle connected to an aspiration pump (913 MityFlex, World Precision Instruments, FL, USA). We hovered the needle closely over the tissue in a circular motion while constantly applying saline solution and monitoring the extent of the aspiration under the stereomicroscope. We continued to perform aspiration until reaching white fibres passing medio-laterally, a feature identifying the corpus callosum. We then continued to aspirate the tissue until we observed fibres passing in the antero-posterior direction. We continued to administer 0.9% saline solution and aspirated it repeatedly until the bleeding stopped.

We held the GRIN lens-electrode assembly (Fig. 3a) using a Proview kit adaptor (Inscopix, CA, USA) attached to the stereotaxic frame. We placed the GRIN lens-electrode assembly over the medial side of the craniotomy so that the lower surface of the lens was at the same height as the edge of the craniotomy. We considered this depth as a reference depth. We raised the lens-electrode assembly, placed it above the centre of the craniotomy and lowered it to 1.25 mm below the reference depth. We applied Kwik-Sil (World Precision Instruments, FL, USA) to cover the space between the edge of the lens-electrode assembly and the brain tissue. After 5 min, the lens-electrode assembly and electrode connector were fixed to the mouse skull by applying the Metabond glue. During this procedure, we covered all implanted screws with glue, being careful not to cover the free end of ground wires connected to ground screws. Once the GRIN lens-electrode assembly was secured to the skull, we soldered the ground wires, connected to the ground screws, to the wires on the connector. We attached the head fixation holder to the skull using glue (Fig. 3b), being careful not to cover the central opening area (Fig. 3d) of the head-fixation holder. Once completed, we removed the mouse from the stereotaxic frame and waited for 3 days for recovery before starting water restriction.

For Fig. 6, while the piece of skull was kept in place, we placed the head fixation holder on top of the mouse skull and fixed it using the Metabond glue (Fig. 5c). During fixation, we covered all implanted screws with the Metabond glue while not covering either the central opening area of the head-fixation holder or the free end of ground wires connected to ground screws. After 15 min, we removed the disconnected piece of the skull using a curved 25G needle. We aspirated tissue in the same manner as described above for Fig. 3 but over larger area (Fig. 5e). Next, we attached the microdrive assembly to a stereotaxic manipulator. The shanks of the silicon probe were tilted to the posterior direction by ~ 40 degrees, measured from the horizontal plane. Once the bleeding from the aspirated tissue fully stopped, we placed the microdrive assembly above the mouse head near the craniotomy (Fig. 5d). We applied a mixture of mineral oil (Sigma-Aldrich M5904) and bone wax (Hospec Medical Equipment & Supplies, FL, USA, MFID: 903) over the shank part of the silicon probe but not within ~ 300 µm from the tips (Fig. 5e,i). We removed all excess saline. Next, we moved the stereotaxic manipulator so that the tips of the silicon probe were placed at the surface of the aspirated tissue and adjusted the position of the microdrive until the four tips of the probe touched the brain at the same time. We attached the microdrive assembly to the skull and the head-fixation holder with the Metabond glue. 15 min later, we turned the screw of the microdrive (Fig. 5d) and inserted the shanks of the silicon probe into the brain until it was approximately 450 µm deep into the brain tissue along the longitudinal axis of the shanks (Fig. 5e,ii.) Using a set of fine forceps attached to the stereotaxic arm, we placed the GRIN lens on top of the craniotomy and then carefully lowered it over the surface of the aspirated tissue as closely to the four tips of the silicon probe as possible without damaging them. Although the placement cannot be precise, this arrangement was expected to result in the placement of the GRIN lens 100–300 µm above the probe tip (Fig. 5e,iii.). We applied Dura-gel mix (Cambridge Neurotech, Cambridge, UK) in the space between the GRIN lens and the probe. After the Dura-gel dried, we applied the Metabond glue to the side of the GRIN lens to attach the GRIN lens to the skull and head-fixation holder (Fig. 5f,i.). Next, we connected ground wires to the ground cannula of the silicon probe. We built a plastic cover around the implant using plastic sheets cut out from weighing boats (Heathrow Scientific, IL, USA, product ID: 120710) to protect the implant (Fig. 5f,ii.) We estimated the anatomical position of the tips of silicon probe by tracking the number of turns of the screw as well as monitoring the changes in the features of LFP while lowering the probe.

For Fig. 8, we performed the same procedures as for Fig. 6 up to tissue aspiration (except the type of head fixation holder was different, and we made the cylindrical shape of aspiration as Fig. 4). Once the bleeding from the aspiration site stopped, we made another hole (Fig. 7di, green) overlapping with the one we made for brain aspiration (Fig. 7di, red). Using a drill bit, we made a mark at 2.9 mm posterior and 1.4 mm right of the bregma. With this mark at its center, we disconnected another circular piece of the skull using the trephine (Fig. 7d,i). Using a drill bit and a pair of forceps, we removed the disconnected piece of the skull. This posterior hole was for inserting the probe (Fig. 7d,iv.). We attached the scaffold to the drive holder (Fig. 7a,iv,ci) and fixed the drive holder to a stereotaxic manipulator. By moving the manipulator, we inserted the GRIN lens into the anterior hole, lowered it 1.25 mm below the reference depth and then applied Kwik-Sil in the same procedures as for Fig. 6 (Fig. 7d,ii,iii). 5 min later, we applied the Metabond glue to the bottom and sides of the shell to attach the scaffold to the skull and head-fixation holder. Once the cement had cured, we attached the back piece (holding the probe connector) to the back of the head-plate using a screw (00–90 x ¼” machine screw) as shown on Fig. 7ci.

We connected ground screws’ wires to the ground cannula of the silicon probe. Before inserting the probe into the brain tissue, we applied a small amount of bone-wax to the exposed part of the silicon probe shanks in the same way as for Fig. 6 (Fig. 5e) while making sure it does not touch the tips. We inserted the probe’s shanks into the brain by turning the driving screw (Fig. 7d,iii,iv). We prepared Dura-gel and applied it over the exposed brain tissue using a bent 25G needle, while being careful to only cover the space between the brain and the lens. Once we completed the surgical procedures, we detached the drive holder from the scaffold and placed a cap to cover the entire implant and then kept it in place using a screw (00–90 x ¼” machine screw) as shown on Fig. 7c,iii.

### Recovery of a silicon probe and a GRIN lens for re-use

At the end of experiment shown in Fig. 8, we deeply anesthetized the mouse and mounted it on the stereotaxic frame. Then, we raised the slider to the topmost position. We attached the drive holder to the stereotaxic frame. We placed it on top of the body to align the screw holes of the body and drive holder. We then inserted two screws to secure the body to the drive holder (as indicated in Fig. 7,iv.). We unscrewed the back piece from the head-plate. We temporarily attached the back piece (glued together with the probe connector) to the drive holder using Blu tack. Finally, we removed the screw connecting the body to the shell (Fig. 7,ii). In this way, we fully detached the body, slider, silicon probe, probe connector and backpiece from the mouse. The shell, GRIN lens, head-plate and head-fixation holder remained on the skull.

We perfusion-fixed the mouse with 4% paraformaldehyde in 0.1 M phosphate buffer. We dissected out the piece of skull that held the remaining parts (the shell, GRIN lens, head plate and head-fixation holder) and incubated it in acetone on a shaker at room temperature, to dissolve/soften Metabond. After 6 h, we were able to easily separate the remaining parts from the skull using forceps. We moved metal pieces (screws and head-fixation holder) to a separate container to avoid damage to the rest of the parts. We incubated these parts in fresh acetone on a shaker at room temperature to further remove Metabond from the parts for at least six more hours. We placed these parts on a clean surface for drying. We removed any remaining piece of Metabond with fine forceps and rinsed the parts with 70% ethanol solution.

We rinsed the recovered silicon probe with ddH_2_O for 30 min, a 10% Decon 90 solution in ddH_2_O for 90 min and then with ddH_2_O for 10 min. Finally, we dipped the silicon probe in a 70% ethanol solution in ddH_2_O. After being dried, the silicon probe is ready to be re-used. We recommend one to check the impedance of individual channels of silicon probe and not to re-use ones in compromised quality. The 3D-printed parts can be often re-used, but it is important to check the absence of damages.

We routinely re-use GRIN lens for multiple implantations. After incubation with acetone described above, we perform additional cleaning. We dip surgical sponges (Sugi, item 30,601, Kettenbach GmbH & Co., Germany) in acetone and wipe the surface of GRIN lens and rinse it with ddH_2_O to ensure that we remove all traces of Metabond from the the side surface of lens. Finally, it is important to inspect each lens using a stereo microscope to check it for any sign of damage to the lens surface, which deteriorates the image quality. We do not re-use a GRIN lens with a sign of damage.

### Calcium imaging

We fixed the head of the mouse by tightly securing the head-fixation holder to a clamp (Fixed-stage positioner designs, https://hhmi.flintbox.com/technologies/fe949d6d-2f87-43ea-b13e-461b558c870d) fixed to the stable parts of the running wheel or treadmill apparatus, depending on the experiments. Next, we moved the objective lens over the head of the mouse. We turned on LED at the minimal power setting and started imaging using the HCImage software (Hamamatsu Photonics K.K., Japan). Next, we adjusted the X–Y position of the objective lens on the top of the implanted GRIN lens by using a fine-adjustment knob (Fig. 2, Table 1) on the microscope assembly so that we observed the circumference of the GRIN lens. Then, we adjusted the Z position of the objective lens so that we observed fluctuations in the fluorescence signal, which corresponds to the activity of neurons or astrocytes. After finding these cells, we adjusted LED power (typically ~ 0.16mW/mm2 at the top surface of the GRIN lens) and image acquisition parameters so that image quality was optimal for each day of recording. We always used an exposure time of 50 ms and an acquisition rate of 20 frames per second. We corrected motion artifacts of the movies using the moco method in ImageJ^24^.

### Electrophysiology

We performed electrophysiological recording using an HS-36 headstage amplifier, the Digital Lynx SX data acquisition system and Cheetah Software 5.7.0 (Neuralynx, Inc., MT, USA). Electrical signal was band-pass filtered at 0.1–9000 Hz for each channel of the electrode. For spike sorting, we used an automated clustering algorithm in klusta software (Rossant et al., 2016). We then manually curated the results using Kwik-gui (https://github.com/cortex-lab/phy/). For analysing sharp-wave ripples, we used Neuralynx CSC filtering tools (Neuralynx, Inc., MT, USA) to apply a band-pass filter of 150–250 Hz to LFP.

## Supplementary Information

Supplementary Video 1.

## Data Availability

The datasets generated during and/or analysed during the current study are available from the corresponding authors on reasonable request.
